# Supplementary material for: Analysis of site and structure specific core fucosylation in liver cirrhosis using exoglycosidase-assisted data-independent LC-MS/MS
Source: Sci Rep. 2021 Dec 2;11:23273. doi: 10.1038/s41598-021-02838-3 (PMC8639754; doi:10.1038/s41598-021-02838-3)
Supplement: Supplementary file 1 — Supplementary Information 1. [file 41598_2021_2838_MOESM1_ESM.pdf]

Analysis of site and structure specific core fucosylation in liver disease progression using exoglycosidase-assisted data-independent LC-MS/MS

Miloslav Sanda[a,d,f], Jaeil Ahn[c], Petr Kozlik[e], Radoslav Goldman[a,b,d]
a Department of Oncology, Lombardi Comprehensive Cancer Center, Georgetown University, Washington, D.C. 20057; b Department of Biochemistry and Molecular & Cellular Biology, Georgetown University, Washington, D.C., 20057; c Department of Biostatistics, Bioinformatics & Biomathematics, Georgetown University Medical Center, Washington, D.C., 20057; d Clinical and Translational Glycoscience Research Center, Georgetown University, Washington, D.C., 20057; e Department of Analytical Chemistry, Faculty of Science, Charles University, Prague, Czech Republic; f Corresponding author email: ms2465@georgetown.edu; tel: +1 202-6876279

Supplemental Figure 1: HILIC chromatography of the glycopeptide of hemopexin treated with fucosidase confirms cleavage of the outer-arm fucose with > 98% efficiency

Supplemental Table 1: Summary of DIA results of selected fucosylated glycopeptides in liver disease

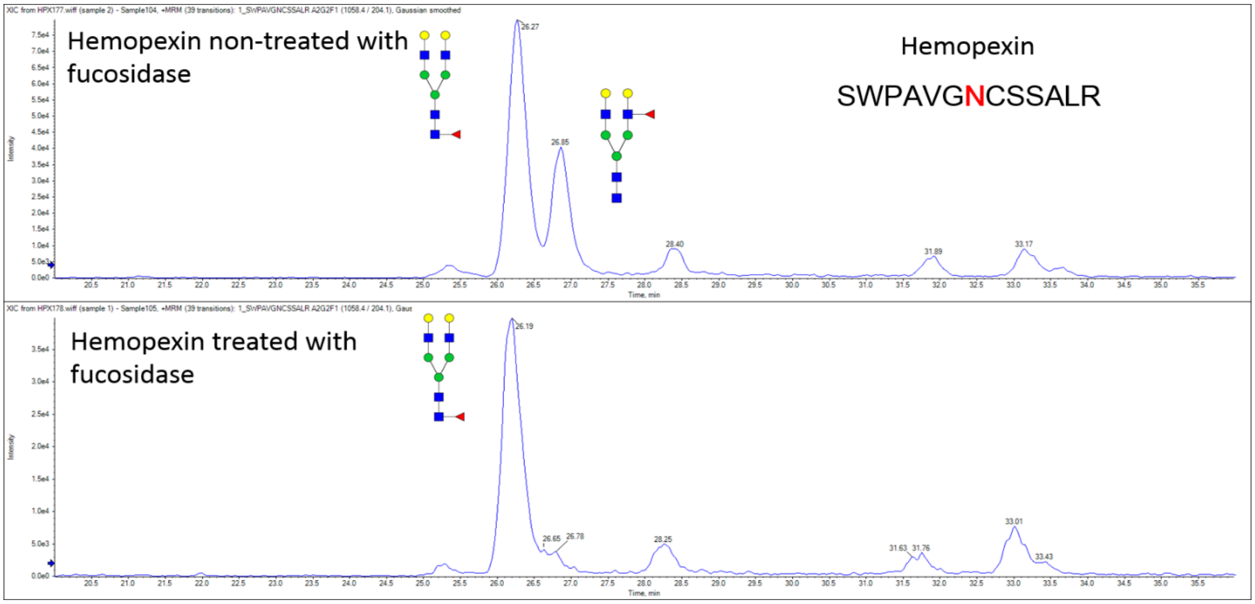

Supplemental Figure 1: HILIC chromatography of the glycopeptide of hemopexin treated with fucosidase confirms cleavage of the outer-arm fucose with > 98% efficiency
